# Functional involvement of endothelial lipase in hepatitis B virus infection

**DOI:** 10.1097/HC9.0000000000000206

**Published:** 2023-08-31

**Authors:** Takayoshi Shirasaki, Kazuhisa Murai, Atsuya Ishida, Kazuyuki Kuroki, Kazunori Kawaguchi, Ying Wang, Souma Yamanaka, Rio Yasukawa, Narumi Kawasaki, Ying-Yi Li, Tetsuro Shimakami, Ariunaa Sumiyadorj, Kouki Nio, Saiho Sugimoto, Noriaki Orita, Hideo Takayama, Hikari Okada, Phuong Doan Thi Bich, Sadahiro Iwabuchi, Shinichi Hashimoto, Mayuko Ide, Noriko Tabata, Satoru Ito, Kouji Matsushima, Hiroshi Yanagawa, Taro Yamashita, Shuichi Kaneko, Masao Honda

**Affiliations:** 1 Department of Clinical Laboratory Medicine, Kanazawa University Graduate School of Medical Sciences, Kanazawa, Japan; 2 Department of Gastroenterology, Kanazawa University Graduate School of Medicine, Kanazawa, Japan; 3 Department of Molecular Pathophysiology, Institute of Advanced Medicine, Wakayama Medical University, Wakayama, Japan; 4 Purotech Bio Inc., Kanagawa, Japan; 5 Division of Molecular Regulation of Inflammatory and Immune Diseases, Research Institute for Biomedical Sciences, Tokyo University of Science, Chiba, Japan

## Abstract

**Background:**

HBV infection causes chronic liver disease and leads to the development of HCC. To identify host factors that support the HBV life cycle, we previously established the HC1 cell line that maintains HBV infection and identified host genes required for HBV persistence.

**Methods:**

The present study focused on endothelial lipase (LIPG), which binds to heparan sulfate proteoglycans (HSPGs) in the cell membrane.

**Results:**

We found HBV infection was impaired in humanized liver chimeric mouse-derived hepatocytes that were transduced with lentivirus expressing short hairpin RNA against LIPG. Long-term suppression of LIPG combined with entecavir further suppressed HBV replication. LIPG was shown to be involved in HBV attachment to the cell surface by using 2 sodium taurocholate cotransporting peptide (NTCP)-expressing cell lines, and the direct interaction of LIPG and HBV large surface protein was revealed. Heparin and heparinase almost completely suppressed the LIPG-induced increase of HBV attachment, indicating that LIPG accelerated HBV attachment to HSPGs followed by HBV entry through NTCP. Surprisingly, the attachment of a fluorescently labeled NTCP-binding preS1 probe to NTCP-expressing cells was not impaired by heparin, suggesting the HSPG-independent attachment of the preS1 probe to NTCP. Interestingly, attachment of the preS1 probe was severely impaired in LIPG knockdown or knockout cells. Inhibitors of the lipase activity of LIPG similarly impaired the attachment of the preS1 probe to NTCP-expressing cells.

**Conclusions:**

LIPG participates in HBV infection by upregulating HBV attachment to the cell membrane by means of 2 possible mechanisms: increasing HBV attachment to HSPGs or facilitating HSPG-dependent or HSPG-independent HBV attachment to NTCP by its lipase activity.

## Graphical abstract

**Figure:**
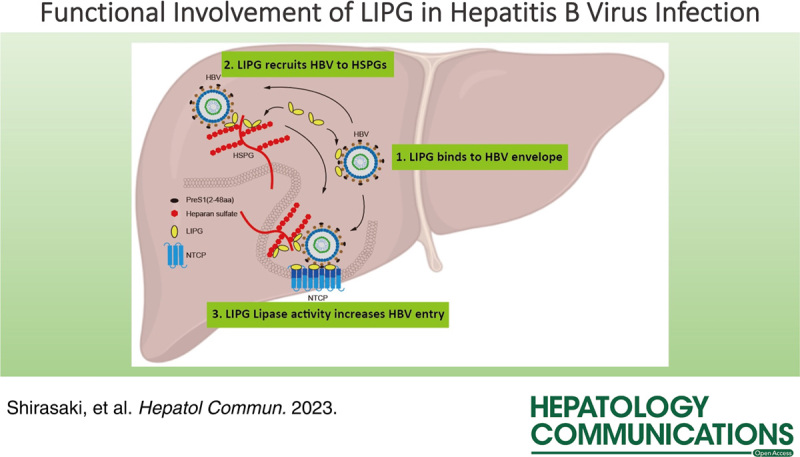

## INTRODUCTION

HBV infection is the major cause of HCC, and 250 million people worldwide are chronic carriers of HBV.^1^ HBV is a DNA virus that has a 3.2-kb-long relaxed circular genome with overlapping open reading frames.^2^ Initially, HBV attaches to hepatocytes by binding to heparan sulfate proteoglycans (HSPGs) in a low-affinity manner and then interacts with its receptor.^3^ In 2012, sodium taurocholate cotransporting peptide (NTCP), which binds to amino acids (aa) 2–48 of the HBV preS1 region, was identified as a novel HBV receptor.^4^ The interaction between HBV and its receptor leads to HBV entry into hepatocytes by means of endocytosis.

Recently, we have established the HC1 cell line in which HBV infection is maintained.^5^ By performing single-cell transcriptome analysis of HC1 cells, we identified host genes that are highly expressed in HBV-infected cells and showed that dedicator of cytokinesis 11 and DENN domain-containing 2A play pivotal roles in the maintenance of HBV in host cells.^5^ In the present study, we focused on another host gene, endothelial lipase (LIPG), which is a member of the triglyceride lipase family and primarily synthesized by vascular endothelial cells.^6^ LIPG is also expressed in tissues including the liver, kidney, lung, testis, and placenta.^7,8^ LIPG plays an important role in HDL metabolism and is also involved in cytokine expression and the lipid composition of cells.^9^ LIPG serves as a bridging molecule between lipoproteins and HSPGs, which leads to the cellular uptake of lipoproteins.^10^ LIPG reportedly hydrolyzes the phosphatidylcholine phospholipids of HDL, after which HDL is selectively taken up by scavenger receptor BI and internalized into hepatocytes.^9^ In HBV infection of hepatocytes, HBV binds to HSPGs first and is then selectively transferred to NTCP and internalized.^11^ Therefore, we reasoned that LIPG plays important functional roles in HBV uptake into hepatocytes. However, little is known about whether LIPG contributes to the HBV life cycle.

## METHODS

### Study approval and ethics statements

All *in vitro* studies were carried out with approval from the ethics committee at Kanazawa University. For human participants, the research protocols were conducted in accordance with both the Declarations of Helsinki and Istanbul, and approved by Human Genome/Gene Analysis Research Ethics Committee of Kanazawa University and its related hospitals. Written informed consent was obtained from all patients.

### Cell lines

HC1, HepG2, HepG2-NTCP-C4, HepG2.2.15, Huh1, Huh6, Huh7, Huh7.5, T5B, HepAD38, HepG2-NTCP-C4-LIPG, Huh7-NTCP-yellow fluorescent protein (YFP), and Huh7-NTCP-LIPG knockout (KO) cells were maintained in Dulbecco’s modified Eagle medium (Thermo Fisher Scientific, Waltham, MA) supplemented with 10% fetal bovine serum (Thermo Fisher Scientific), 1% l-glutamine (Thermo Fisher Scientific), and 1% penicillin/streptomycin (Thermo Fisher Scientific) in a humidified atmosphere of 5% CO_2_ at 37°C. Humanized liver chimeric mouse-derived hepatocytes (PXB cells) were purchased from PhoenixBio (Hiroshima, Japan).

### Establishment of HepG2-NTCP-C4-LIPG, Huh7-NTCP-YFP, and Huh7-NTCP-LIPG KO cells

For the preparation of HepG2-NTCP-C4-LIPG cells, a lentiviral transfer plasmid encoding *LIPG* was created by PCR amplifying an *LIPG* cDNA plasmid as a template using the forward primer 5′-TTAGAATTCATGAGCAACTCCGTTC-3′ with an *Eco*RI restriction site and reverse primer 5′-AATTCTAGATCACTTGTCATCGTCATCCTTGTAGTCGGGAAGCTCCACAGTG-3′ with an *Xba*I restriction site. The PCR product was digested with the above enzymes and ligated into the similarly digested pLVSIN-CMV Pur vector (Takara Bio, Otsu, Japan) to obtain the pLVSIN-CMV-Flag tagged *LIPG* vector. Viral particles were generated by transfecting plated Lenti-X 293T cells (Takara Bio) with the pLVSIN-CMV Pur vector or pLVSIN-CMV-Flag-tagged *LIPG* vector along with Lentiviral High Titer Packaging Mix (Takara Bio) using the FuGENE-HD Transfection Reagent (Promega, Madison, WI). The supernatant fluids harvested at 72 hours were filtered through a 0.22-μm syringe filter and used to transduce cells. At 72 hours after transduction, these cells were treated with 5 mg/mL puromycin for antibiotic selection. We used antibiotic-resistant bulk cell populations for experiments to avoid clonal biases.

For the preparation of Huh7-NTCP-YFP cells, the NTCP-YFP expression vector pENNTCPY was constructed using NTCP-YFP DNA in which YFP DNA from pEYFP-N1 (Takara Bio USA, San Jose, CA) was fused to the carboxy terminal site of NTCP cDNA synthesized from human liver total RNA (Takara Bio USA) downstream from the CMV chicken β-actin (ACTB) promoter of pEB Multi-Neo derivative (Fujifilm Wako Pure Chemical Corp., Osaka, Japan). Huh7-NTCP-YFP cells were obtained from Huh7 cells by transfection with pENNTCPY.

Huh7-NTCP-LIPG KO cells were prepared from Huh7 cells by transfection with LIPG sgRNA (CRISPR677191_SGM; Thermo Fisher Scientific), Cas9 protein (Takara Bio), and a gRNA transfection kit (GenomONE-GE; Ishihara Sangyo, Osaka, Japan). LIPG editing sites were confirmed by Sanger sequencing.

### Reagents and antibodies

Heparin, heparinase, orlistat, and entecavir (ETV) were purchased from R&D Systems (Minneapolis, MN). The LIPG inhibitor GSK-264220A was purchased from Tocris Bioscience (Minneapolis, MN). Myrcludex B was purchased from Cosmo Bio (Tokyo, Japan). Primary antibodies to DYKDDDDK (FLAG) (#14793), ACTB (#4970), and GAPDH (#5147) were from Cell Signaling Technology (Danvers, MA); LIPG (ab24447) was from Abcam (Cambridge, UK); GFP (A-11122) was from Thermo Fisher Scientific; HBV core (HBP-023-9) was from Austral Biologicals (San Ramon, CA); and HBV preS1 was from GenScript (Tokyo, Japan). ELISA kit for LIPG (MBS2501814) was purchased from MyBioSource (San Diego, CA).

### Knockdown (KD) of *LIPG* by short hairpin RNA (shRNA)

shRNA targeting *LIPG* (target sequence: 5′-TTACACGGATGCGGTCAATAA-3′), which was cloned into a lentiviral-based pLKO.1-puro expression vector, was purchased from Sigma-Aldrich. Lentiviral particles were produced in packaging cells (293FT cells) by co-transfection with packaging plasmids. Stable *LIPG* knockdown (KD) cells were selected using the puromycin selectable marker.

### RNA interference

Small interfering RNA (siRNA) targeting *LIPG* and negative control siRNA were obtained from Sigma-Aldrich. siRNA transfection was performed using Lipofectamine RNAiMAX Transfection Reagent (Invitrogen).

### Quantification of HBV DNA and covalently closed circular DNA (cccDNA) by quantitative PCR (qPCR)

HBV DNA was extracted from the cells using a DNeasy Blood & Tissue Kit (QIAGEN). The extracted DNA (250 ng) was treated for 30 minutes at 37°C with 25 U T5 exonuclease (New England BioLabs, Ipswich, MA), and treated for 5 minutes at 95°C for enzyme heat inactivation. HBV DNA and covalently closed circular DNA (cccDNA) levels were quantified with qPCR MasterMix Plus Low ROX (Nippon Gene, Tokyo, Japan) using a specific HBV DNA probe (5′-FAM/TATCGCTGG/ZEN/ATGTGTCTGCGGCGT/3IBFQ-3′) and cccDNA probe (5′-FAM-CTGTAGGCATAAATTGGT-MGB-3′).

### qRT-PCR

Total RNA was isolated using a GenElute Mammalian Total RNA Miniprep Kit (Sigma-Aldrich), and cDNA was synthesized using a High-Capacity cDNA Reverse Transcription Kit (Applied Biosystems, Carlsbad, CA). qRT-PCR was performed on the 7500 Real-Time PCR System (Applied Biosystems) using 5′-GCTCTGTATCGGGAGGCCTTA-3′ and 5′-TGAGTGCTGTATGGTGAGGAGAA-3′ as primers and 5′-FAM-AGTCTCCGGAACATT-MGB-3′ as a probe for pregenomic RNA (pgRNA). The primer pairs and probes for *LIPG* and *ACTB* were obtained from the TaqMan assay reagents library.

### SDS-polyacrylamide gel electrophoresis and immunoblotting

The cells were washed in PBS and lysed in a RIPA Lysis Buffer (EMD Millipore, Burlington, MA) containing Complete Protease Inhibitor Cocktail (Roche Applied Science, Penzberg, Germany). Cell membrane and cytoplasmic proteins of Huh7-NTCP-YFP cells were fractionated using an EzSubcell Extract Kit (ATTO, Tokyo, Japan). Western blotting was performed with standard methods. The membranes were blocked in Blocking One solution (Nacalai Tesque, Kyoto, Japan). The ChemiDoc Imaging System (Bio-Rad, Hercules, CA) was used for visualization.

### Immunoprecipitation assay

Cell lysates were incubated with rabbit IgG, rabbit anti-Flag, or rabbit anti-HA antibodies overnight at 4°C. Immunoprecipitation was performed using Protein G Mag Sepharose (GE Healthcare, Little Chalfont, UK). The beads were washed in lysis buffer and eluted in SDS sample buffer. The reaction mixtures were analyzed by immunoblotting.

### PreS1 binding assay

The preS1 probe, N-terminally myristoylated and C-terminally TAMRA-conjugated 2–48 aa of the preS1 region, was synthesized (GenScript, Piscataway, NJ). Huh7-NTCP-YFP cells were seeded at 1.0 × 10^5^ cells/well in a 4-chamber slide, and at 16 hours later, they were transfected with siRNA at a final concentration of 10 nM using Lipofectamine RNAiMAX Transfection Reagent (Invitrogen). At 48 hours after transfection, the cells were treated with the preS1 probe at a final concentration of 40 nM and incubated at 37°C for 30 minutes. After incubation, the cells were fixed with 4% paraformaldehyde for 30 minutes at room temperature and then permeabilized with PBS containing 0.3% Triton X-100 and 1% bovine serum albumin for 1 hour at room temperature. The cells were stained using PLUS Antifade Mounting Medium with DAPI (Vector Laboratories, Newark, CA), and the fluorescent signal was observed with fluorescence microscopy.

### Establishment of the HiBiT assay system for binding of HSPGs and HBV particles

Recently, we have reported a novel HBV infection monitoring system using a luminescent 11-amino acid reporter, the high-affinity subunit of nano-luciferase binary technology (HiBiT), cloned upstream of a genotype C HBV genome, to generate recombinant cell culture-derived virus (HiBiT-HBVcc).^12^ Whole cell lysates of Huh7-NTCP-YFP and HepG2-NTCP-C4 Lenti-Ctrl or Lenti-LIPG cells were added to the plate of a Human Heparan Sulfate ELISA Kit (Elabscience, Houston, TX), and incubated for 90 minutes at 37°C with or without recombinant human LIPG protein (Abcam). After incubation, the plate was washed 3 times with wash buffer. HiBiT-HBVcc (10,000 copies) was added to the plate and incubated for 60 minutes at 37°C. Heparin was used as a negative control at a final concentration of 100 IU/mL. The plate was washed 3 times with wash buffer. To measure the HBV particles attached to HSPGs, HiBiT activity was determined by using the Nano Glo HiBiT Lytic Detection System (Promega) with the GloMax-Multi+Detection System (Promega).

### Statistical analysis

Unless noted otherwise, all between-group comparisons were carried out by 1-way or 2-way ANOVA or a 2-sided *t* test. Calculations were made using Prism 7 software (GraphPad Software, La Jolla, CA).

## RESULTS

### LIPG is upregulated in HBV-positive HC1 cells and regulates HBV infection

**FIGURE 1:**
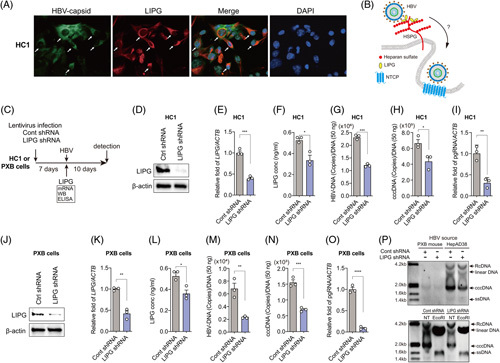
LIPG is upregulated in HBV-positive HC1 cells and regulates HBV infection. (A) Coimmunofluorescence staining of HBV capsid using an anti-HBV core antibody and LIPG using an anti-LIPG antibody in HC1 cells. DAPI was used to stain nuclei. (B) Conceptual schematic of the hypothesis. (C) Schematic of the experimental design. (D) Immunoblot analysis of cellular LIPG and ACTB in Cont shRNA-transduced HC1 cells and LIPG shRNA-transduced HC1 cells at 7 days after transduction. (E) qRT-PCR analysis of *LIPG* mRNA in Cont shRNA-transduced HC1 cells and LIPG shRNA-transduced HC1 cells at 7 days after transduction. The results were normalized to those of *ACTB*. (F) LIPG concentrations in the medium of Cont shRNA-transduced HC1 cells and LIPG shRNA-transduced HC1 cells at 7 days after transduction. (G) qPCR analysis of HBV DNA in Cont shRNA-transduced HC1 cells and LIPG shRNA-transduced HC1 cells at 10 days after HBV infection. (H) qPCR analysis of cccDNA in Cont shRNA-transduced HC1 cells and LIPG shRNA-transduced HC1 cells at 10 days after HBV infection. (I) qRT-PCR analysis of pgRNA transcripts in Cont shRNA-transduced HC1 cells and LIPG shRNA-transduced HC1 cells at 10 days after HBV infection. The results were normalized to those of *ACTB*. (J) Immunoblot analysis of cellular LIPG and ACTB in Cont shRNA-transduced PXB cells and LIPG shRNA-transduced PXB cells at 7 days after transduction. (K) qRT-PCR analysis of *LIPG* mRNA in Cont shRNA-transduced PXB cells and LIPG shRNA-transduced PXB cells at 7 days after transduction. The results were normalized to those of *ACTB*. (L) LIPG concentrations in the medium of Cont shRNA-transduced HC1 cells and LIPG shRNA-transduced HC1 cells at 7 days after transduction. (M) qPCR analysis of HBV DNA in Cont shRNA-transduced PXB cells and LIPG shRNA-transduced PXB cells at 10 days after HBV infection. (N) qPCR analysis of cccDNA in Cont shRNA-transduced PXB cells and LIPG shRNA-transduced PXB cells at 10 days after HBV infection. (O) qRT-PCR analysis of pgRNA transcripts in Cont shRNA-transduced PXB cells and LIPG shRNA-transduced PXB cells at 10 days after HBV infection. The results were normalized to those of *ACTB*. (P: upper) Southern blot analysis of Cont shRNA-transduced PXB cells and LIPG shRNA-transduced PXB cells after 10 days infection with HBV derived from PXB mice or HepAD38 cells. (P: lower) Southern blot analysis of NT or *Eco*RI-digested DNA of Cont shRNA-transduced PXB cells and LIPG shRNA-transduced PXB cells after 10 days infection with HBV derived from HepAD38 cells. Data shown represent means ± SEM from 3 independent experiments. Statistical testing was done by a 2-tailed unpaired *t* test. ****p < 0.0001, ***p < 0.001, **p < 0.01. Abbreviations: HSPG, heparan surface proteoglycan; LIPG, endothelial lipase; NT, nontreated; PXB, humanized liver chimeric mouse-derived hepatocytes.

Single-cell transcriptome analysis of HC1 cells enabled us to identify host genes that are highly expressed in HBV-infected cells.^5^ In addition to previously reported host factors, dedicator of cytokinesis 11 and DENN domain-containing 2A,^5^ we found that LIPG was upregulated in HBV-positive HC1 cells (Supplemental Table S1, http://links.lww.com/HC9/A456). Immunofluorescence staining of HBV capsid and LIPG showed that both proteins were coexpressed in HC1 cells (Figure 1A). LIPG contains a heparin-binding domain and binds to HSPGs on the plasma membrane.^13^ In HBV infection of hepatocytes, HBV binds to HSPGs first, and is then selectively transferred to NTCP and internalized (Figure 1B).^11^ Therefore, we hypothesized that LIPG participates in HBV entry by interacting with HSPGs and HBV (Figure 1B). To determine the effect of LIPG on HBV infection, HC1 cells were transduced with LIPG-specific shRNA (LIPG shRNA) or nontargeting shRNA (Cont shRNA) by using a lentivirus expression system (Figure 1C). LIPG protein and mRNA levels in cell lysate were significantly reduced in HC1 cells transduced with LIPG shRNA compared with control shRNA (Figure 1D, E). In addition, the concentration of LIPG in the culture medium of LIPG KD HC1 cells was significantly decreased compared to control cells (Figure 1F). At 10 days after HBV infection (Figure 1C), LIPG KD HC1 cells showed a significant reduction of HBV DNA, cccDNA, and pgRNA transcripts compared with control cells (Figure 1G–I). Next, we assessed whether these findings also occurred in PXB cells. PXB cells were similarly transduced with LIPG or control shRNA by using a lentivirus expression system and then infected with HBV (Figure 1C). LIPG mRNA and protein levels and concentration in medium were significantly reduced in PXB cells by LIPG shRNA compared with control shRNA (Figure 1J–L). LIPG KD PXB cells showed a significant reduction of HBV DNA, cccDNA, and pgRNA transcripts (Figure 1M–O) compared with control cells. The reduction of cccDNA was also confirmed by Southern blotting (Figure 1P). Collectively, these results suggest that LIPG is a host factor that regulates HBV infection.

### LIPG increases the attachment of HBV to permissive cell lines

**FIGURE 2:**
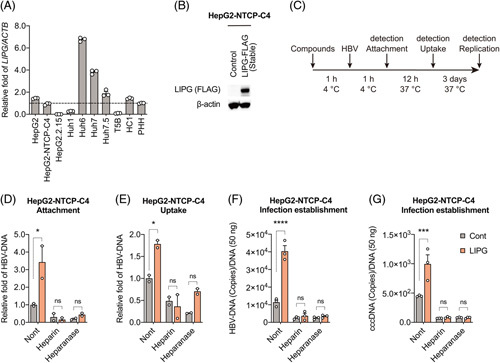
LIPG increases the attachment of HBV to permissive cell lines. (A) qRT-PCR analysis of *LIPG* mRNA in HepG2, HepG2-NTCP-C4, HepG2.2.15, Huh1, Huh6, Huh7, T5B, HC1, and PXB cells. The results were normalized to those of *ACTB*. (B) Immunoblot analysis of Flag-tagged LIPG and ACTB in HepG2-NTCP-C4-Cont and HepG2-NTCP-C4-LIPG cells. (C) Schematic of the experimental design. (D) qPCR analysis of HBV DNA in HepG2-NTCP-C4-Cont and HepG2-NTCP-C4-LIPG cells at 1 hour after HBV inoculation at 4°C in the presence or absence of heparin and heparinase. (E) qPCR analysis of HBV DNA in HepG2-NTCP-C4-Cont and HepG2-NTCP-C4-LIPG cells at 12 hours after reincubation at 37°C. (F) qPCR analysis of HBV DNA in HepG2-NTCP-C4-Cont and HepG2-NTCP-C4-LIPG cells at 3 days after reincubation at 37°C. (G) qPCR analysis of cccDNA in HepG2-NTCP-C4-Cont and HepG2-NTCP-C4-LIPG cells at 3 days after reincubation at 37°C. Data shown represent means ± SEM from 3 (A, F, and G) or 2 independent experiments (D and E). Statistical testing was done by 2-way ANOVA with Tukey multiple comparison test. ****p < 0.0001, ***p < 0.001, *p < 0.05, not significant (ns). Abbreviations: LIPG, endothelial lipase; NTCP, sodium taurocholate cotransporting peptide.

To determine how LIPG regulates HBV infection, we examined its effect on the entry of HBV into host cells and conducted an attachment and uptake assay. We compared the expression levels of LIPG in various hepatoma cell lines and found that its expression was comparable between HC1 and HepG2-NTCP-C4 cells^14^ (Figure 2A). HepG2-NTCP-C4 cells overexpressing FLAG-tagged LIPG were established by using a lentivirus expression system. The abundant expression of FLAG-tagged LIPG protein was observed in HepG2-NTCP-C4-LIPG cells (Figure 2B). For the attachment and uptake assay, as control compounds, heparin, which inhibits HBV attachment to target cells,^3^ and heparinase, which is a mammalian endo-β-D-glucuronidase that can cleave the glycosaminoglycan heparan sulfate side chains of HSPGs, were used.^15^ The cells were pretreated with the compounds for 1 h, after which HBV was adsorbed to the cells for 1 hour at 4°C and then washed off prior to the cultures being shifted to 37°C for 12 hours (Figure 2C). Inoculation with HBV was followed by culturing the cells in normal growth medium for an additional 3 days until HBV DNA and cccDNA were detected (Figure 2C). LIPG overexpression increased HBV attachment (Figure 2D), uptake (Figure 2E), and infection establishment (HBV DNA and cccDNA) (Figure 2F, G) in comparison to control cells. As expected, heparin and heparinase-reduced HBV attachment, uptake, and infection establishment in control cells and these compounds also reduced the LIPG-mediated increase of HBV attachment, uptake, and infection establishment (Figure 2D–G). We examined whether LIPG had a positive effect on the parental HepG2 cell line; however, LIPG overexpression did not increase the attachment of HBV to HepG2 cells (Supplemental Figure S1, http://links.lww.com/HC9/A456).

### LIPG suppression decreases HBV attachment to permissive cell lines

**FIGURE 3:**
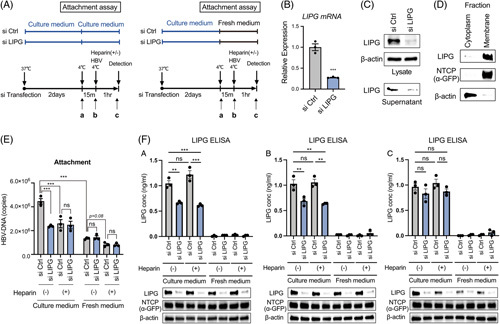
Suppression of LIPG decreases the attachment of HBV to permissive cell lines. (A: left) Schematic of the experimental design with culture medium. (A: right) Schematic of the experimental design with changing from culture medium to fresh medium. (B) qRT-PCR analysis of *LIPG* mRNA in control siRNA (si Ctrl)-transfected Huh7-NTCP-YFP cells and LIPG siRNA (si LIPG)-transfected Huh7-NTCP-YFP cells at 2 days after transfection. The results were normalized to those of *ACTB*. (C) Immunoblot analysis of cellular LIPG and ACTB and supernatant LIPG in control siRNA (si Ctrl)-transfected Huh7-NTCP-YFP cells and LIPG siRNA (si LIPG)-transfected Huh7-NTCP-YFP cells at 2 days after transfection. (D) Immunoblot analysis of LIPG and NTCP in the cytoplasmic and membrane fractions. (E) qPCR analysis of HBV DNA in control siRNA (si Ctrl)-transfected Huh7-NTCP-YFP cells and LIPG siRNA (si LIPG)-transfected Huh7-NTCP-YFP cells at 1 hour after HBV inoculation at 4°C. The experiments were performed in culture medium or fresh medium in the presence or absence of heparin. (F) Measurement of LIPG concentrations in culture medium by ELISA, and immunoblotting of LIPG and NTCP in the membrane fraction of control siRNA (si Ctrl)-transfected Huh7-NTCP-YFP cells and LIPG siRNA (si LIPG)-transfected Huh7-NTCP-YFP cells at the beginning of the attachment assay (a), at the time of HBV infection after 15 minutes (b), and at the time of detection after 1 hour (c), as indicated in the experimental design (A). Data shown represent means ± SEM from 3 independent experiments. Statistical testing was done by a 2-tailed unpaired *t* test (A, B, and C) or 2-way ANOVA with Tukey multiple comparison test (D and E). ****p < 0.0001, ***p < 0.001, *p < 0.05. Abbreviations: Ctrl, control; LIPG, endothelial lipase; ns, not significant.

To examine the effect of the loss of LIPG function on HBV attachment, we used Huh7 cells derived from a cell line that expresses a relatively high amount of LIPG (Figure 2A). Huh7-NTCP-YFP cells expressing the NTCP-YFP fusion protein on the cell surface were established. NTCP expression was confirmed by a fluorescent signal on the cell membrane, while HBV infection was confirmed by super-resolution confocal microscopy (Supplemental Figure S2, http://links.lww.com/HC9/A456) and Southern blotting (Supplemental Figure S3, http://links.lww.com/HC9/A456). Huh7-NTCP-YFP cells were transfected with siRNA against LIPG at 2 days before the attachment assay (Figure 3A). The suppression of LIPG mRNA and protein in the cell lysate and supernatant was confirmed by qRT-PCR and western blotting, respectively (Figure 3B, C). LIPG was mainly present in the membrane fraction, with very low levels in the cytoplasmic fraction (Figure 3D).

For the attachment assay, the cells were incubated at 4°C for 15 minutes, and then, HBV was adsorbed to the cells for 1 hour at 4°C (Figure 3A). HBV DNA levels were significantly lower in LIPG KD cells than in control cells (Figure 3E). Heparin reduced HBV attachment to control cells (Figure 3E). Interestingly, when the culture medium was changed to fresh medium during the attachment assay, HBV attachment was generally reduced and the differences between LIPG KD cells and control cells were lost (Figure 3E). Conversely, restoring the reduced levels of LIPG using medium derived from non-LIPG KD cells rescued the decrease of HBV attachment (Supplemental Figure S4, http://links.lww.com/HC9/A456). Thus, the presence of LIPG in the medium was a critical factor for HBV attachment.

Heparin treatment reduced LIPG levels in the cell lysate (Supplemental Figure S5, http://links.lww.com/HC9/A456), assuming heparin absorbed LIPG from HSPGs on the cell surface and increased heparin binding to LIPG was detected in the supernatant (Supplemental Figure S5, http://links.lww.com/HC9/A456).

To examine the molecular events during the attachment assay, LIPG concentrations in the medium were measured by ELISA, and the levels of LIPG and NTCP in the membrane fraction of control siRNA (si Ctrl)-transfected Huh7-NTCP-YFP cells and LIPG siRNA (si LIPG)-transfected Huh7-NTCP-YFP cells were evaluated by immunoblotting at the beginning of the attachment assay (a), at the time of HBV infection after 15 minutes (b), and at the time of detection after 1 hour (c), as indicated in the experimental design in Figure 3A. LIPG concentration in the medium and LIPG and NTCP levels in the membrane were generally the same at the time of (a) and (b). A significantly lower concentration of LIPG was observed in the medium of LIPG KD cells than in the medium of non-LIPG KD cells, and the fresh medium contained no LIPG. At the time of (c), at 1 hour after HBV infection, there was no increase of LIPG concentration in the fresh medium. Unexpectedly, LIPG content in the medium of LIPG KD cells increased and there was no significant difference in LIPG concentration between LIPG KD and non-LIPG KD cells. Heparin treatment and fresh medium decreased the membrane level of LIPG within 1 hour, but there were still differences in LIPG levels between non-LIPG KD and LIPG KD cells (Figure 3F-c). Thus, changing the culture medium to fresh medium started to decrease the level of membrane LIPG, but it could not reach equilibrium with the LIPG in the medium within 1 hour, as no trace of LIPG was detected in the medium. Collectively, LIPG content in the medium was a critical factor for HBV attachment in these experimental conditions.

We further confirmed the effect of LIPG on the attachment of HDV that possesses the HBV surface protein on its envelope. Hepatitis delta virus attachment was significantly suppressed in heparin-treated control cells and LIPG KD cells (Supplemental Figure S6, http://links.lww.com/HC9/A456).

### LIPG interacts directly with HBV large surface protein

**FIGURE 4:**
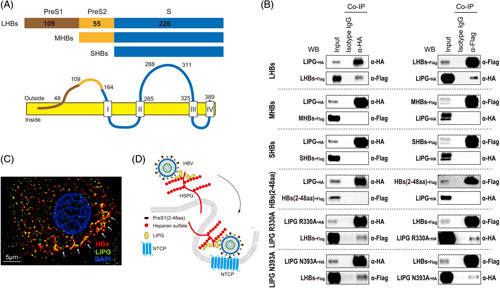
LIPG interacts directly with LHBs. (A) Structure of LHBs. (B) Coimmunoprecipitation assays of LIPG and its related mutants (R330A and N393A) with LHBs, MHBs, SHBs, and the preS1 (2–48 aa) domain. (C) Coimmunofluorescence staining of LHBs using an anti-LHBs antibody and LIPG using an anti-LIPG antibody in Huh7 cells. DAPI was used to stain nuclei. (D) Conceptual schematic of the hypothesis. Abbreviations: LHB, large surface protein; LIPG, endothelial lipase; MHB, middle surface protein.

As LIPG was involved in HBV attachment, we hypothesized that it could bind directly to HBV. To confirm the direct binding of LIPG to large surface proteins (LHBs), we performed an immunoprecipitation assay. HA-tagged LIPG (LIPG-HA) and Flag-tagged LHBs (LHBs-Flag) (Figure 4A) were overexpressed in Huh7 cells, and LIPG was immunoprecipitated using an anti-HA antibody. Interestingly, we found that LHBs coprecipitated with LIPG (Figure 4B). Conversely, when LHBs was immunoprecipitated using an anti-Flag antibody, LIPG coprecipitated with LHBs (Figure 4B). Super-resolution microscopy showed the colocalization of LIPG and LHBs on the cell surface (Figure 4C). LIPG did not bind to HBV middle surface protein (MHBs) or small surface protein (SHBs) (Figure 4B). These results suggest the possible involvement of the PreS1 region of LHBs for LIPG binding; however, LIPG did not bind to the NTCP-binding peptide of preS1 (2–48 aa) (Figure 4B).

As for LIPG, the noncleaved mutant R330A^16^ and N-glycosylation mutant N393A^17^ bound to LHBs (Figure 4B), supporting the specific binding of LIPG to LHBs. Thus, LIPG potentially facilitates HBV binding to HSPGs through a direct interaction with LHBs. These results also show that LIPG may not mask the NTCP-binding site of LHBs (Figure 4D).

### LIPG increases the binding of HSPGs and HBV particles

**FIGURE 5:**
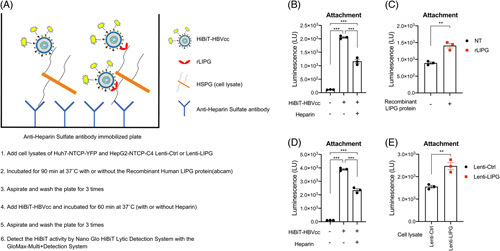
Establishment of the HiBiT assay system for the binding of HSPGs and HBV particles. (A) Schematic representation of the HiBiT assay system for the binding of HSPGs and HiBiT-HBVcc. (B) Binding of HSPGs derived from Huh7-NTCP-YFP cells and HiBiT-HBVcc. Heparin treatment reduced binding. (C) The addition of recombinant LIPG protein (100 μg/well) significantly increased the binding of HSPGs and HiBiT-HBVcc. The values for heparin treatment were subtracted from the shown values. (D) Binding of HSPGs derived from HepG2-NTCP-C4 cells transduced with Lenti-Ctrl and HiBiT-HBVcc. Heparin treatment reduced binding. (E) HSPGs derived from HepG2-NTCP-C4 cells transduced with Lenti-LIPG showed significantly higher binding to HiBiT-HBVcc than HSPGs from control cells transduced with Lenti-Ctrl. The values for heparin treatment were subtracted from the shown values. Statistical testing was done by 2-way ANOVA with Tukey multiple comparison test. ***p < 0.001, **p < 0.01. Abbreviations: Ctrl, control; HSPG, heparan surface proteoglycan; LIPG, endothelial lipase.

We next examined whether LIPG increased the binding of HSPGs and HBV particles. For this assay, we used a recombinant cell culture-derived virus labeled with a luminescent 11-amino acid reporter (HiBiT-HBVcc) (Figure 5A).^12^ HSPGs in cells were captured by an anti-heparin sulfate antibody that was immobilized on the plate, and then HiBiT-HBVcc was added for binding. As the source of HSPGs, lysates of Huh7-NTCP-YFP cells were used, and significant luminescent signals were observed (Figure 5B). Heparin decreased these signals, and the values were used as background signals. The addition of recombinant LIPG protein (100 μg/well) significantly increased the binding of HSPGs and HiBiT-HBVcc (Figure 5C). Similarly, as the source of HSPGs, lysates of HepG2-NTCP-C4 cells transduced with Lenti-Ctrl were used (Figure 5D). HSPGs derived from HepG2-NTCP-C4 cells transduced with Lenti-LIPG showed significant binding to HiBiT-HBVcc compared to HSPGs from control cells transduced with Lenti-Ctrl (Figure 5E). These data indicate that LIPG protein or HSPGs associated with LIPG increase the binding of HSPGs and HBV particles.

### LIPG is involved in the attachment of the HBV preS1 probe to NTCP in permissive cells

**FIGURE 6:**
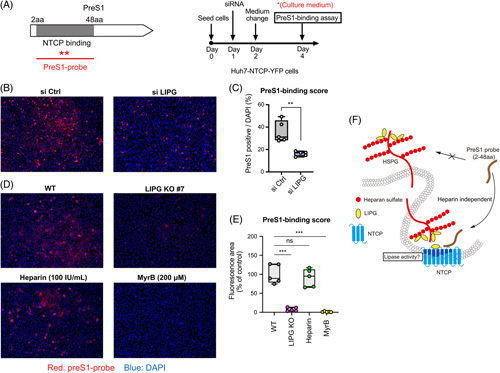
LIPG is involved in the attachment of the HBV preS1 probe to NTCP in permissive cells. (A: left) NTCP- and preS1 probe-binding region in the preS1 region. The aa 2–48 domain in the preS1 region binds to NTCP to facilitate viral entry. (A: right) Schematic of the experimental design. (B) HBV preS1-mediated attachment to cells was examined in control siRNA (si Ctrl)-transfected Huh7-NTCP-YFP cells and LIPG siRNA (si LIPG)-transfected Huh7-NTCP-YFP cells exposed for 30 minutes at 37°C to a TAMRA-labeled preS1 probe. Red and blue signals indicate the preS1 probe and nucleus, respectively. (C) PreS1 binding score. (D: upper left and upper right) HBV preS1-mediated attachment to cells was examined in Huh7-NTCP and Huh7-NTCP-LIPG KO cells exposed for 30 minutes at 37°C to a TAMRA-labeled preS1 probe. (D: lower left and lower right) HBV preS1-mediated attachment to cells was examined in Huh7-NTCP-YFP cells exposed for 30 minutes at 37°C to a TAMRA-labeled preS1 probe in the presence or absence of 100 IU/mL heparin or 200 μM myrcludex B. (E) PreS1 binding score. (F) Conceptual schematic of the hypothesis. Boxplots: center line, median; box limits, 25th to 75th percentiles; whiskers, minimum to maximum. Data shown represent means ± SEM from 5 independent experiments. Statistical testing was done by a 2-tailed unpaired *t* test (C) or 2-way ANOVA with Tukey multiple comparison test (E). ***p < 0.001, **p < 0.01. Abbreviations: Ctrl, control; KO, knockout; LIPG, endothelial lipase; WT, wild type.

We next examined whether LIPG was involved in the attachment of the fluorescently labeled preS1 probe (2–48 aa) to NTCP in Huh7-NTCP-YFP cells (Figure 6A). As LIPG binds to LHBs, except through the preS1 region (2–48 aa), and the heparin-binding region of LHBs could be the antigenic loop in preS2.^18^ LIPG might be irrelevant for the binding of the preS1 probe (2–48 aa) to NTCP. However, an attachment assay showed that LIPG KD with siRNA significantly reduced the fluorescent signal of the preS1 probe (2–48 aa) compared with control siRNA (Figure 6B, C), which was confirmed by using LIPG KO cells (Figure 6D, E and Supplemental Figure S7, http://links.lww.com/HC9/A456). Heparin treatment did not affect the attachment of the preS1 probe (2–48 aa) to Huh7-NTCP-YFP cells (Figure 6D, E), confirming the previous report that the preS1 region is irrelevant for heparin binding.^18^ Importantly, myrcludex B, equivalent to an unlabeled preS1 probe (2–48 aa), competitively inhibited the attachment of the fluorescently labeled preS1 probe (Figure 6D, E). These results suggest that LIPG might be involved in preS1 probe binding to NTCP independent of heparin binding or direct binding to preS1 (Figure 6F).

### Lipase activity of LIPG participates in the attachment of HBV to permissive cells

**FIGURE 7:**
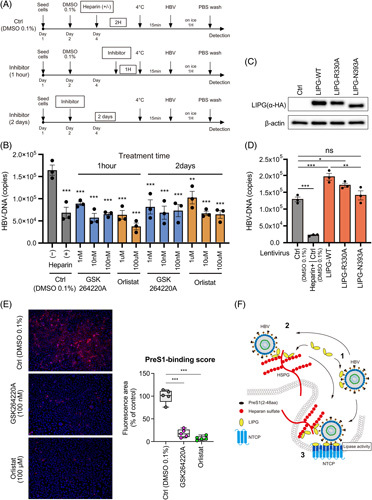
Lipase activity of LIPG participates in the attachment of HBV to permissive cells. (A) Schematic of the experimental design. (B) qPCR analysis of HBV DNA in GSK-264220A- or orlistat-treated Huh7-NTCP-YFP cells at 1 hour after HBV inoculation at 4°C. (C) Immunoblotting analysis of the overexpression of LIPG and its related mutants (R330A and N393A) in Huh7-NTCP-YFP cells. (D) Effect of the overexpression of LIPG and its related mutants (R330A and N393A) in Huh7-NTCP-YFP cells on HBV attachment. The assay was performed at 1 hour after HBV inoculation at 4°C. (E: left) HBV preS1-mediated attachment to cells was examined in Huh7-NTCP-YFP cells exposed for 30 minutes at 37°C to a TAMRA-labeled preS1 probe in the presence or absence of GSK-264220A or orlistat. (C: right) PreS1 binding score. (F) Conceptual schematic of the hypothesis. 1, LIPG and free HBV. 2, Interaction of HBV and HSPGs through LIPG. 3, Lipase activity of LIPG and HBV attachment. Boxplots: center line, median; box limits, 25th to 75th percentiles; whiskers, minimum to maximum. Data shown represent means ± SEM from 3 (B) or 5 independent experiments (C). Statistical testing was done by 2-way ANOVA with Tukey multiple comparison test. ***p < 0.001, **p < 0.01.Abbreviation: Ctrl, control; LIPG, endothelial lipase; WT, wild type.

LIPG has lipase activity that catalyzes the hydrolysis of the phosphatidylcholine phospholipids of HDL.^6^ Phosphatidylcholine is one of the major components of biological membranes. Therefore, we hypothesized that LIPG might have some function that increased the attachment of the preS1 probe to NTCP. We used 2 LIPG inhibitors, GSK-264220A and orlistat. Pretreatment of Huh7-NTCP-YFP cells with these LIPG inhibitors for either 1 hour or 2 days significantly decreased the attachment of HBV to Huh7-NTCP-YFP cells (Figure 7A, B).

Furthermore, we examined the effect of mutant LIPG with defective lipase activity on HBV attachment. The overexpression of wild-type LIPG, LIPG R330A, and LIPG N339A was confirmed (Figure 7C), and interestingly, mutant LIPG lost the ability to enhance HBV attachment (Figure 7D).

We next examined whether GSK-264220A and orlistat could inhibit the attachment of the preS1 probe (2–48 aa) to Huh7-NTCP-YFP cells. Both compounds significantly inhibited the attachment of the preS1 probe (2–48 aa) (Figure 7E). Therefore, the lipase activity of LIPG participates in the attachment of HBV to NTCP (Figure 7F).

### Long-term suppression of LIPG in PXB cells substantially reduces HBV replication

**FIGURE 8:**
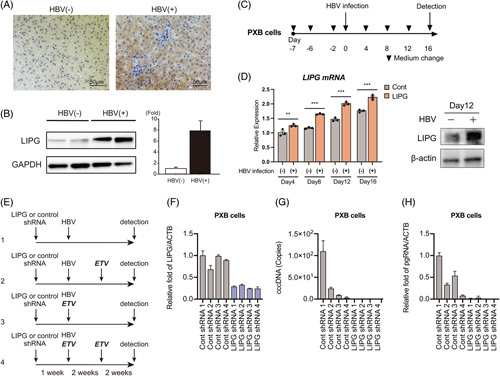
LIPG expression is upregulated by HBV infection. (A: left) Immunohistochemical staining of LIPG in liver tissue from a normal liver patient. (A: right) Immunohistochemical staining of LIPG in liver tissue from a patient with chronic hepatitis B. (B: left) Immunoblot analysis of LIPG and GAPDH in liver tissue from normal liver patients and patients with chronic hepatitis B. (B: right) Relative intensity. (C) Schematic of the experimental design. (D: left) qRT-PCR analysis of *LIPG* mRNA in mock-infected PXB cells and HBV-infected PXB cells at 16 days after HBV infection. The results were normalized to those of *ACTB*. Data shown represent means ± SEM from 3 independent experiments. Statistical testing was done by a 2-tailed unpaired *t* test. *p < 0.05. (D: right) Immunoblot analysis of LIPG and ACTB in mock-infected PXB cells and HBV-infected PXB cells at 12 days after HBV infection. (E) Schematic of the experimental design. (F) qRT-PCR analysis of *LIPG* mRNA in Cont shRNA-transduced PXB cells and LIPG shRNA-transduced PXB cells at 5 weeks after shRNA transduction in the presence or absence of ETV. The results were normalized to those of *ACTB*. (G) qPCR analysis of cccDNA in Cont shRNA-transduced PXB cells and LIPG shRNA-transduced PXB cells at 4 weeks after HBV infection in the presence or absence of ETV. (H) qRT-PCR analysis of pgRNA transcripts in Cont shRNA-transduced PXB cells and LIPG shRNA-transduced PXB cells at 4 weeks after HBV infection in the presence or absence of ETV. The results were normalized to those of *ACTB*. Data shown represent means ± SEM from 3 independent experiments. Abbreviations: ETV, entecavir; LIPG, endothelial lipase; PXB, humanized liver chimeric mouse-derived hepatocytes.

Immunohistochemical staining of LIPG in normal liver showed that LIPG was expressed mainly in the cytoplasm and membrane of hepatocytes according to a protein expression database (The Human Protein Atlas; https://www.proteinatlas.org/). Interestingly, immunohistochemical staining and western blotting analysis showed the increased expression of LIPG in the liver of patients with chronic hepatitis B compared with normal liver patients (Figure 8A, B). Moreover, HBV infection of PXB cells significantly increased LIPG transcription over 16 days (Figure 8C, D), which was confirmed by western blotting (Figure 8D). Thus, HBV infection upregulates LIPG expression at the mRNA and protein levels.

We next examined the effect of LIPG KD on cccDNA levels in long-term cultures of PXB cells after HBV infection. PXB cells were transduced with LIPG or control shRNA by using a lentivirus expression system and these cells were then infected with HBV (Figure 8E). PXB cells were cultured for a further 4 weeks with regular changes of the culture medium. ETV was added at the time of HBV infection and/or at 2 weeks after HBV infection (Figure 8E). LIPG expression was significantly reduced in PXB cells over 4 weeks by LIPG shRNA compared with control shRNA (Fig. 8F). There was no obvious survival defect in LIPG KD PXB cells. After 4 weeks of HBV infection, LIPG KD reduced cccDNA and pgRNA levels to below the limit of detection (141 copies/2.5 ng DNA) (Figure 8G, H). ETV treatment alone reduced cccDNA levels, but they were still detectable after 4 weeks (Figure 8G). The combination of ETV treatment and LIPG KD reduced cccDNA and pgRNA levels to below the limit of detection (Figure 8G, H).

## DISCUSSION

To elucidate the host factors required for HBV persistence, we established a new cell line, HC1, which was derived from a chronic hepatitis B patient complicated with HCC.^5^ This cell line is unique and useful because it potentially contains the host factors required for the establishment of long-term HBV infection. In this study, we showed that LIPG plays important roles in the HBV life cycle. LIPG is an HSPG-binding protein that plays a key role in the uptake of HDL into hepatocytes; HDL attaches to LIPG on HSPGs and is then selectively taken up by scavenger receptor BI and internalized.^9^ This process resembles HBV entry into hepatocytes: HBV first binds to HSPGs and is then selectively taken up by NTCP and internalized.^11^ Dane particles are reported to be 42 nm in size, while subviral particles are 22 nm.^19^ The size of HDL varies and is distributed from 7 to 30 nm.^20^ Therefore, we reasoned that LIPG might also play important roles in HBV entry.

We showed that LIPG was involved in HBV infection using HC1 cells and PXB cells (Figure 1). We clearly demonstrated that LIPG played a role in HBV attachment using HepG2-NTCP-C4 cells (Figure 2) and Huh7-NTCP-YFP cells (Figure 3); however, LIPG did not affect the attachment of HBV to the parental HepG2 cell line. Therefore, LIPG functioned as an enhancer of HBV-specific receptors such as NTCP or possibly undefined HBV receptors.

By using an attachment assay, we showed that LIPG in the culture medium was an important regulator of HBV attachment (Figure 3 and Supplemental Figure S4, http://links.lww.com/HC9/A456). The attachment of HBV was reduced in LIPG-free fresh medium, and this decreased attachment of HBV was rescued by restoring the level of LIPG in the culture medium. We found that the decreased level of LIPG in the culture medium was almost recovered at 1 hour after the addition of HBV (Figure 3F-c). Although HBV infection increased LIPG expression (Figure 8D), the recovery of LIPG in the culture medium may not be due to HBV infection because it occurred during the 1-hour attachment period. It could be speculated that the free form of LIPG binds to HBV and the LIPG-bound HBV can then attach to the cell membrane. Binding activity might be high if HBV was associated with multiple LIPGs, and low if HBV was associated with few LIPGs. Therefore, in medium with a low level of LIPG, less HBV bound to the membrane and the concentration of unbound LIPG associated with HBV increased, thereby increasing the concentration of LIPG in the medium. However, in medium with a high level of LIPG, more HBV bound to the membrane and the concentration of unbound LIPG associated with HBV decreased and then the level of LIPG in the medium tended to be decreased (Figure 3F-C).

LIPG is secretory protein, and secreted LIPG binds to HSPGs on the cell surface. In this sense, LIPG may serve as a carrier of HBV by binding to HBV and carrying it to HSPGs. In this study, we showed that free LIPG and membrane-associated LIPG increased the binding of HSPGs and HBV particles by using the HiBiT assay system (Figure 5).

We also demonstrated the direct interaction of LIPG and LHBs by immunoprecipitation and super-resolution confocal microscopy (Figure 4B, C). LIPG bound directly to LHBs, but did not bind to MHBs or SHBs (Figure 4B). Therefore, the preS1 region could be responsible for LIPG binding. However, an NTCP-binding peptide of preS1 (2–48 aa) could not bind to LIPG. Thus, LIPG may not mask HBV for NTCP binding (Figure 4D). Detailed alanine scanning of the preS1 region in the context of the role of LHBs for LIPG binding is underway.

We also showed that the attachment of the preS1 probe (2–48 aa) was substantially decreased in LIPG KD and KO cells (Figure 6). As heparin treatment did not affect the attachment of the preS1 probe (2–48 aa), but an unlabeled preS1 probe (myrcludex B) blocked its attachment, the effect of LIPG on the binding of the preS1 probe (2–48 aa) to NTCP was heparin-independent (Figure 6B–F). These results indicate that the lipase activity of LIPG might be involved in the attachment of the preS1 region of HBV to NTCP (Figure 6F).

We showed that LIPG lipase activity plays an important role in HBV attachment (Figure 7A, B), and the increased attachment of HBV in the presence of LIPG was abolished by LIPG mutants with impaired lipase activity (Figure 7C, D). HBV might be connected to the lipoprotein metabolic pathways to induce its uptake into the liver. Our study confirmed the findings of a previous study showing that orlistat prevents HBV infection by targeting an early step in the virus life cycle.^21^

Our observations indicate that there are 3 possible steps by which LIPG increases HBV entry. First, free LIPG binds to HBV and then LIPG-bound HBV attaches to HSPGs on the cell membrane as the second step. This process might be independent of its lipase activity because 2 lipase defective mutants (R330A and N393A)^16,17^ could bind to LHBs (Figure 4B). However, the lipid composition of HBV surface proteins resembles that of HDL^22^, so LIPG might affect free HBV particles directly. Future studies should assess how LIPG changes the structure of HBV particles biochemically. Third, the HSPG-LIPG-HBV complex would be internalized through NTCP, with the lipase activity of LIPG necessary for this process. LIPG might affect the cell membrane to increase HBV attachment, and we showed that this process might be used for HSPG-dependent and HSPG-independent HBV entry (Figures 6F and 7F).

LIPG expression is reportedly induced by the inflammatory cytokines IL-1β and TNF α in endothelial cells.^8^ However, the regulation of LIPG expression in hepatocytes has not been characterized. Interestingly, LIPG expression was increased in liver tissue from patients with chronic hepatitis B compared with healthy controls (Figure 8A, B). We demonstrated that HBV infection increased LIPG expression in PXB cells (Figure 8C, D). Further studies are needed to reveal the regulation of LIPG expression by HBV infection in hepatocytes.

Finally, long-term suppression of LIPG in PXB cells substantially reduced HBV replication, and its combination with ETV administration provided the promising suppression of cccDNA and pgRNA (Figure 8E–H). In conclusion, we have obtained preliminary data suggesting the possible involvement of LIPG in HBV replication in cells as well as in HBV entry. These findings suggest that LIPG may be a potential target for the development of new anti-HBV drugs.

## FUNDING INFORMATION

This research was partially supported by the Japan Agency for Medical Research and Development (grant numbers JP22fk0310514, JP22fk0210112, JP22fk0210081, JP22ek0210154, JP22fk0210095, and JP22fk0210073) and a Grant-in-Aid for Scientific Research (A) (grant number JP21H04823).

## CONFLICTS OF INTEREST

The authors have no conflicts to report.

## Supplementary Material
