# Supplementary material for: Functional involvement of endothelial lipase in hepatitis B virus infection
Source: Hepatol Commun. 2023 Aug 31;7(9):e0206. doi: 10.1097/HC9.0000000000000206 (PMC10476801; doi:10.1097/HC9.0000000000000206)
Supplement: Supplementary file 1 [file hc9-7-e0206-s001.docx]

**Supporting Information for**

**Functional involvement of endothelial lipase in hepatitis B virus infection**

Wang^1^, Souma Yamanaka^1^, Rio Yasukawa^1^, Narumi Kawasaki^1^, Ying-Yi Li^2^, Tetsuro Shimakami^2^, Kouki Nio^2^, Saiho Sugimoto^2^, Noriaki Orita^2^, Hideo Takayama^2^, Hikari Okada^2^, Phuong Doan Thi Bich^2^, Sadahiro Iwabuchi^3^, Shinichi Hashimoto^3^, Mayuko Ide^4^, Noriko Tabata^4^, Satoru Ito^4^, Kouji Matsushima^5^, Hiroshi Yanagawa^4^, Taro Yamashita^2^, Shuichi Kaneko^2^, and Masao Honda^1,2^

^1^ Department of Clinical Laboratory Medicine, Kanazawa University Graduate School of

Health Medicine, Kanazawa, Japan

^2^ Department of Gastroenterology, Kanazawa University Graduate School of Medicine,

Kanazawa, Japan

^3^ Department of Molecular Pathophysiology, Institute of Advanced Medicine, Wakayama Medical University, Wakayama, Japan

^4^ Purotech Bio Inc., Kanagawa, Japan

^5^ Division of Molecular Regulation of Inflammatory and Immune Diseases. Research

Institute for Biomedical Sciences, Tokyo University of Science, Chiba, Japan

**Supplemental Table 1**

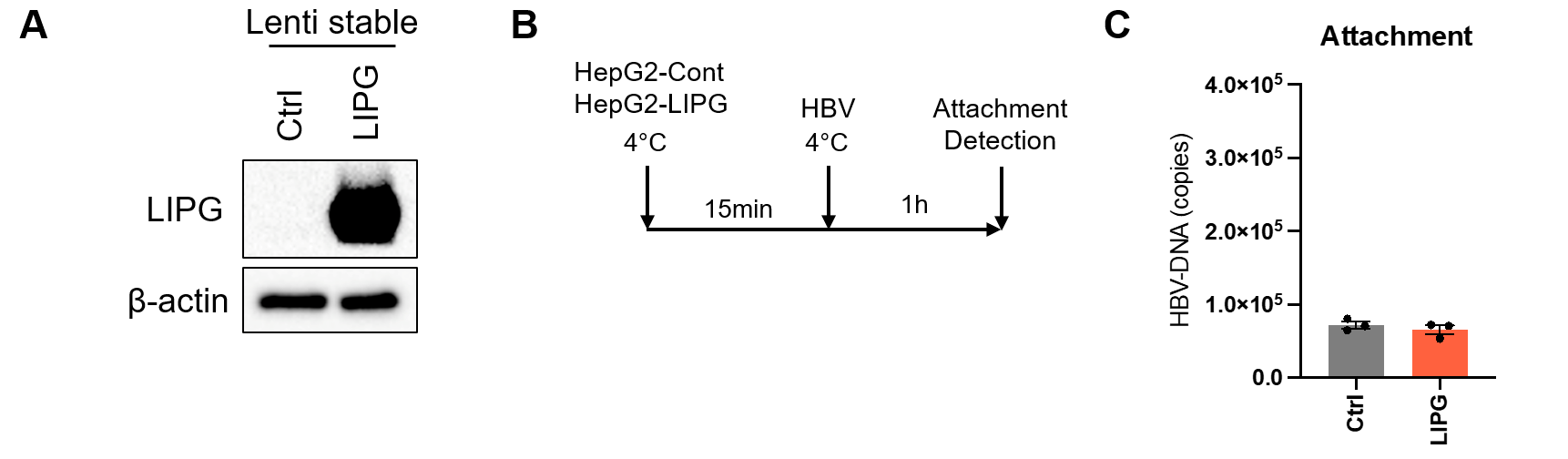


**Supplemental Figure 1**

**Effect of LIPG on non-HBV permissive cell lines.** (A) Immunoblot analysis of Flag-tagged LIPG and ACTB in HepG2-Cont and HepG2-LIPG cells. (B) Schematic of the experimental design. (C) qPCR analysis of HBV-DNA or cccDNA in HepG2-Cont and HepG2-LIPG cells at 1 h after HBV inoculation at 4°C.


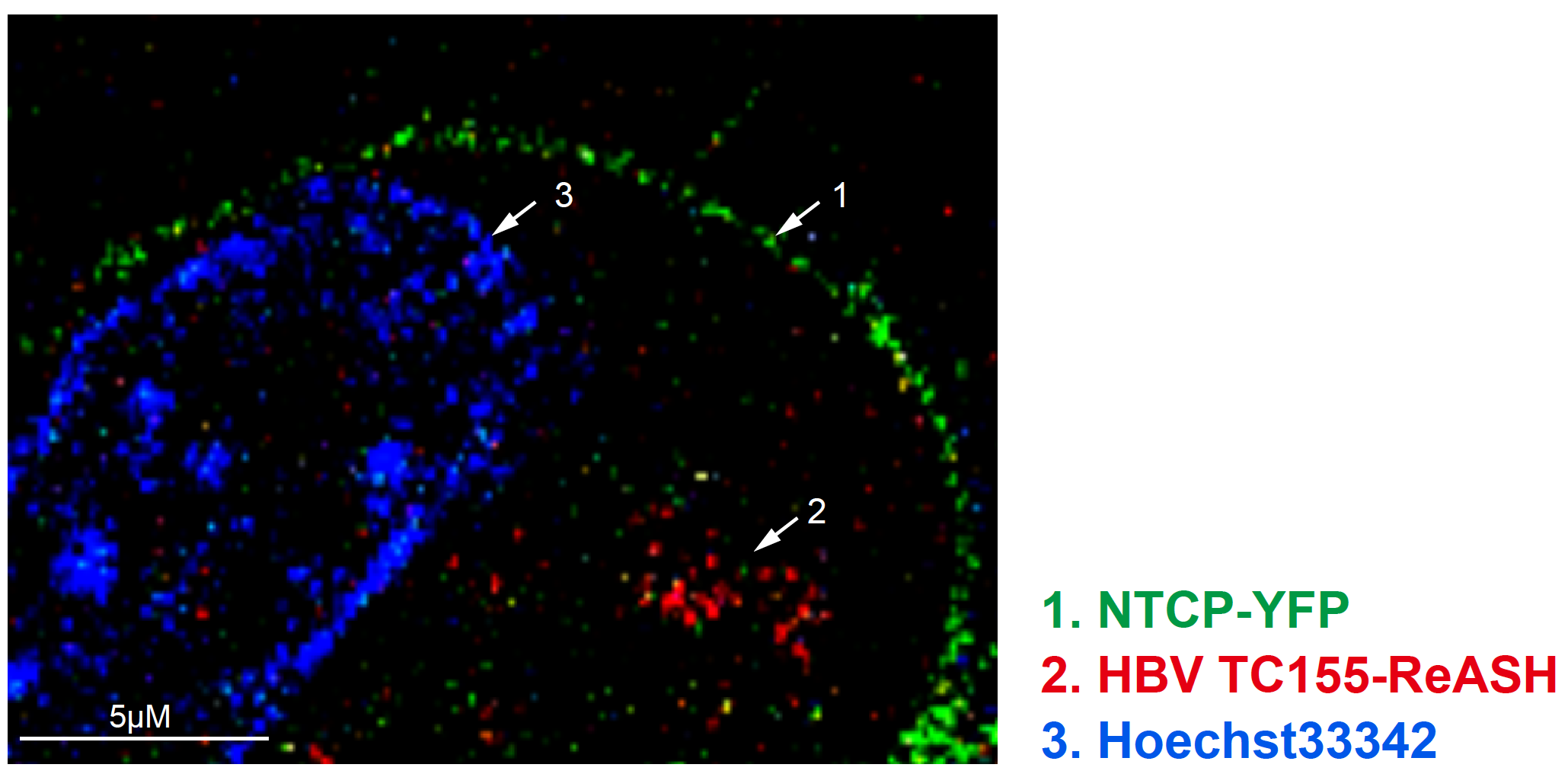


**Supplemental Figure 2**

**Establishment of Huh7-NTCP-YFP cells.** HBV TC155-ReASH has a tetracysteine (TC) tag (C-C-P-G-C-C) at the 155^th^ capsid aa and is labeled with the ReASH fluorescent dye. Huh7-NTCP-YFP cells were infected with fluorescently-labeled HBV TC155-ReASH. An HM-1000 super-resolution microscope was utilized to detect the co-localization of capsid with YFP (NTCP). Hoechst 33342 was used to stain nuclei.


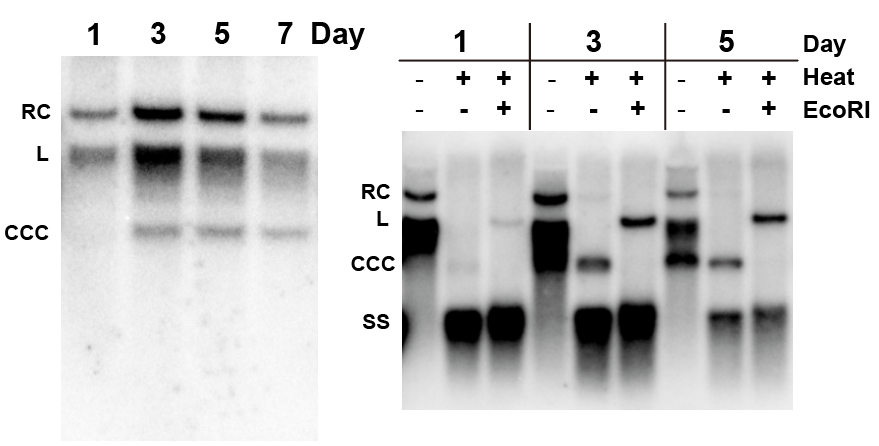


**Supplemental Figure 3**

**Southern blot analysis of Huh7-NTCP-YFP cells infected with HBV.** Huh7-NTCP-YFP cells were infected with HBV prepared from HepAD38 cells and Southern blot analysis was performed on days 1, 3, 5, and 7 after infection. cccDNA was detected from 3 to 7 days after infection. Heat denaturation shifted RC and L DNA to SS DNA, but cccDNA was stable. *Eco*RI digestion reduced cccDNA levels. ccc: covalently closed circular DNA; L: linear DNA; RC: relaxed circular DNA; SS: single stranded DNA.

**
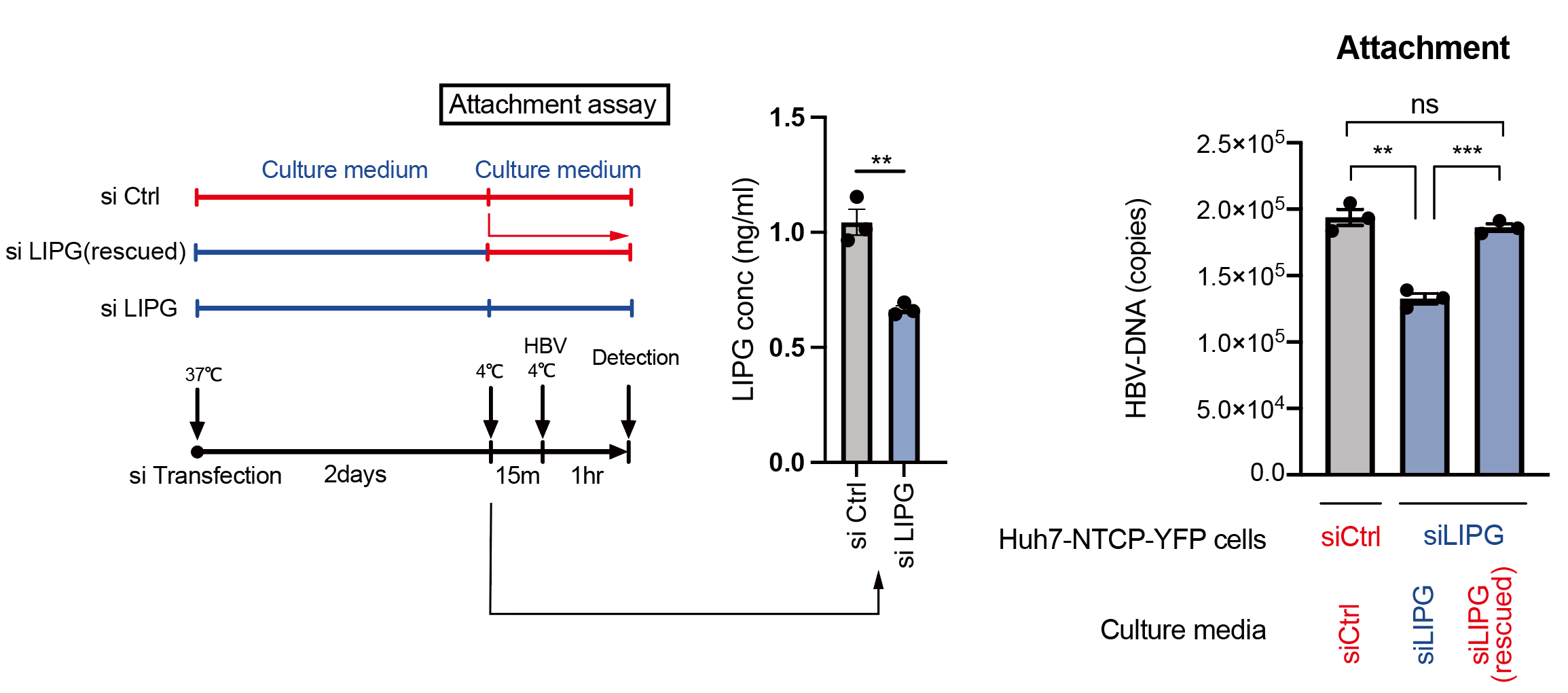
**

**Supplemental Figure 4**

**Rescue of HBV attachment by restoring downregulated LIPG in culture medium.** Decreased LIPG in culture medium of siRNA-transfected Huh7-NTCP-YFP cells was confirmed by ELISA. Culture medium of siLIPG-transfected cells was replaced with that of siCtrl-transfected cells to restore the decreased levels of LIPG in the culture medium. An HBV attachment assay was performed as described (left). Decreased HBV attachment was rescued by restoring the concentration of LIPG in the culture medium (right).


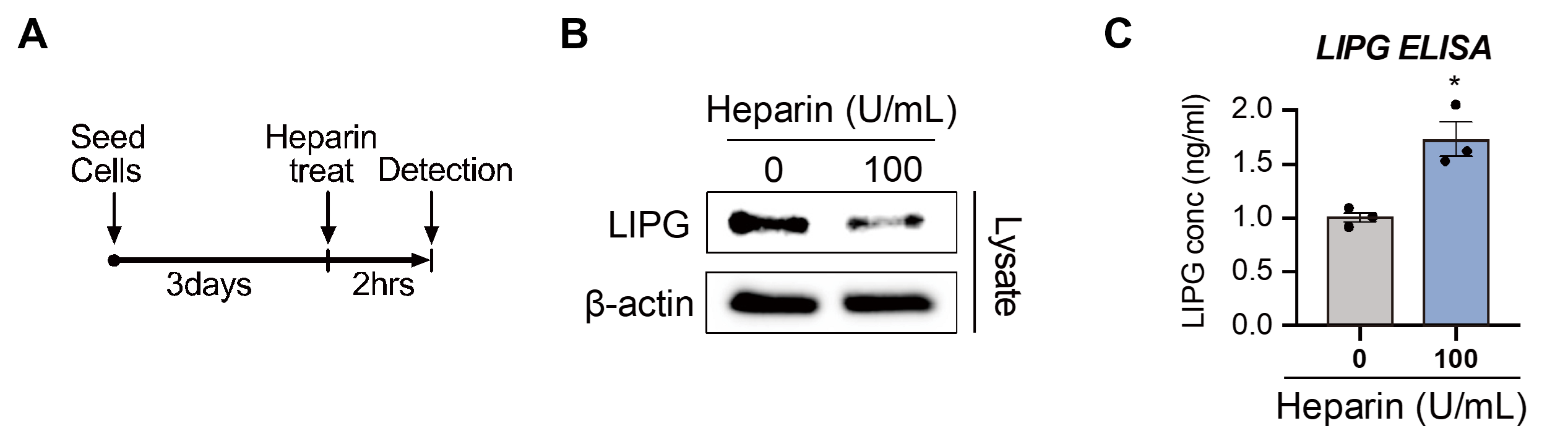


**Supplemental Figure 5**

**Heparin competes out LIPG from the cell surface.** (A) Huh7-NTCP-YFP cells were treated with 100 U/mL heparin for 2 h. (B) Immunoblot analysis of LIPG and ACTB in Huh7-NTCP-YFP cells with or without heparin treatment. (C) LIPG concentrations in the culture medium of Huh7-NTCP-YFP cells with or without heparin treatment.


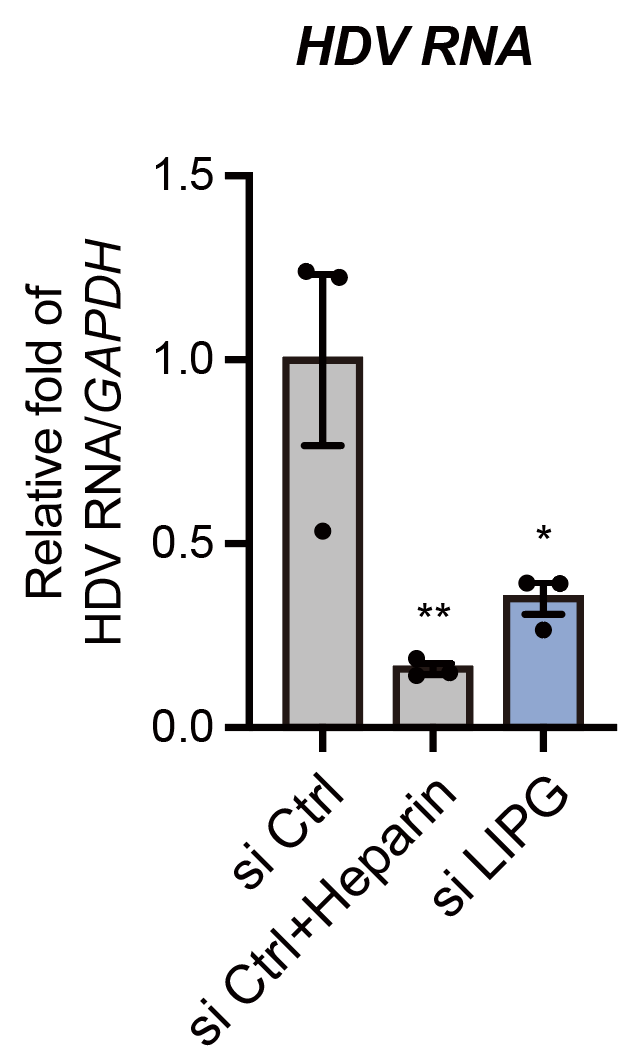


**Supplemental Figure 6**

**HDV attachment assay.** An HDV attachment assay was performed as for the HBV attachment assay with culture medium (Figure 3A). Preparation of HDV and the assay protocol are described in Supplemental Material and Methods. HDV attachment was significantly suppressed in heparin-treated control cells and LIPG KD cells. Data shown represent means ± SEM. ***p* < 0.01, **p* < 0.05.

**
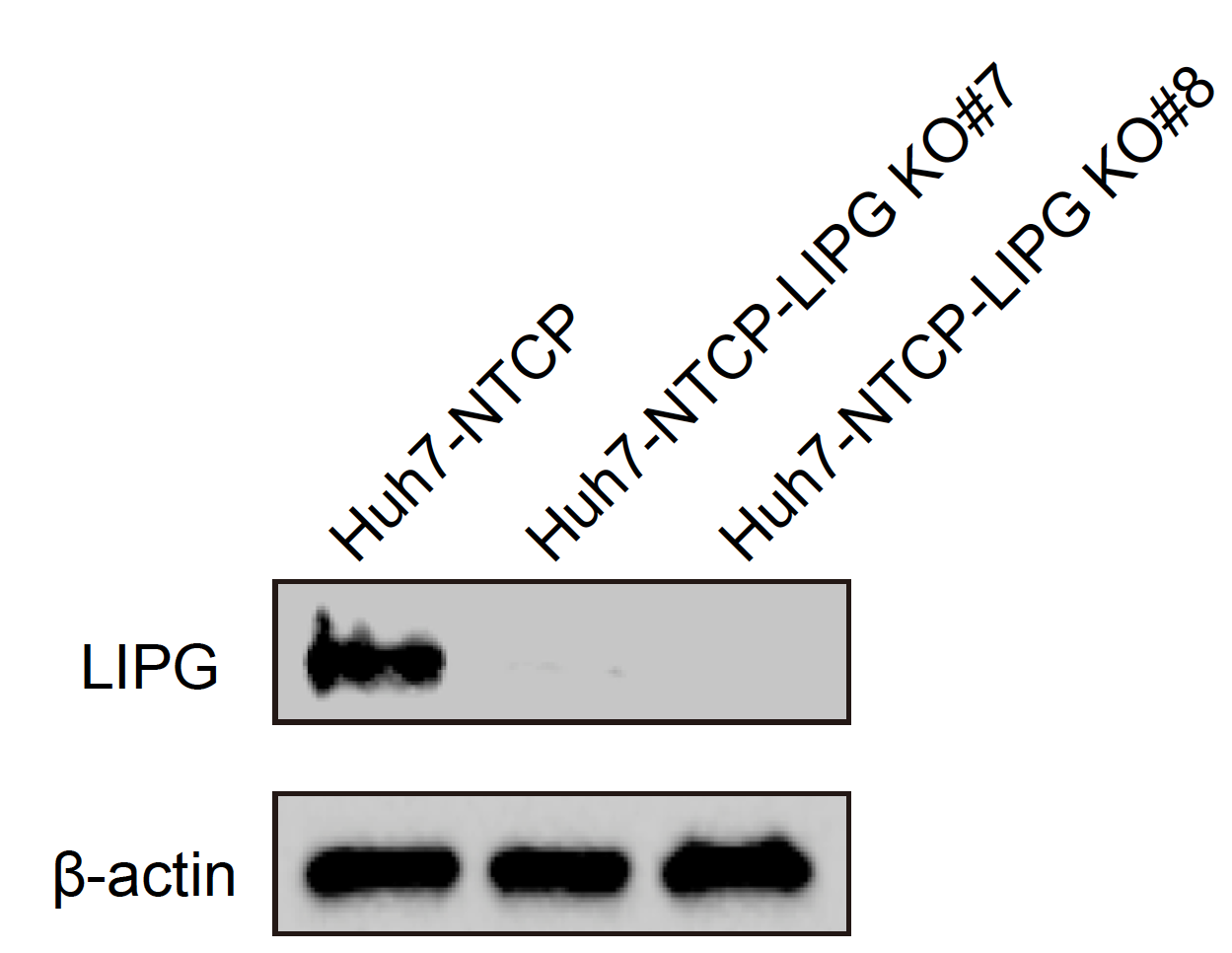
**

**Supplemental Figure 7**

**Establishment of Huh7-NTCP-LIPG KO cells.** Immunoblot analysis of LIPG and ACTB in Huh7-NTCP cells and Huh7-NTCP-LIPG KO cells (knockout clonal cells #7 and #8).
